# Circulating MyomiRs as Potential Biomarkers to Monitor Response to Nusinersen in Pediatric SMA Patients

**DOI:** 10.3390/biomedicines8020021

**Published:** 2020-01-26

**Authors:** Silvia Bonanno, Stefania Marcuzzo, Claudia Malacarne, Eleonora Giagnorio, Riccardo Masson, Riccardo Zanin, Maria Teresa Arnoldi, Francesca Andreetta, Ornella Simoncini, Anna Venerando, Cinzia Gellera, Chiara Pantaleoni, Renato Mantegazza, Pia Bernasconi, Giovanni Baranello, Lorenzo Maggi

**Affiliations:** 1 Neurology IV–Neuroimmunology and Neuromuscular Diseases Unit, Fondazione IRCCS Istituto Neurologico Carlo Besta, Via Celoria 11, 20133 Milan, Italy; claudia.malacarne@istituto-besta.it (C.M.); eleonora.giagnorio@istituto-besta.it (E.G.); francesca.andreetta@istituto-besta.it (F.A.); ornella.simoncini@istituto-besta.it (O.S.); pia.bernasconi@istituto-besta.it (P.B.); lorenzo.maggi@istituto-besta.it (L.M.); 2 PhD Program in Neuroscience, University of Milano-Bicocca, via Cadore 48, 20900 Monza, Italy; 3 Developmental Neurology Unit, Fondazione IRCCS Istituto Neurologico Carlo Besta, Via Celoria 11, 20133 Milan, Italy; riccardo.masson@istituto-besta.it (R.M.); riccardo.zanin@istituto-besta.it (R.Z.); mariateresa.arnoldi@istituto-besta.it (M.T.A.); chiara.pantaleoni@istituto-besta.it (C.P.); giovanni.baranello@istituto-besta.it (G.B.); 4 Unit of Medical Genetics and Neurogenetics, Fondazione IRCCS Istituto Neurologico Carlo Besta, Via Celoria 11, 20133 Milan, Italy; cinzia.gellera@istituto-besta.it (C.G.); anna.venerando@istituto-besta.it (A.V.); 5 The Dubowitz Neuromuscular Centre, UCL NIHR GOSH Biomedical Research Centre, Great Ormond Street Institute of Child Health, London WC1N 1EH, UK

**Keywords:** spinal muscular atrophy, nusinersen, myomiRNAs, biomarkers

## Abstract

Spinal muscular atrophy (SMA) is an autosomal recessive disorder caused by mutations in survival motor neuron (SMN) 1 gene, resulting in a truncated SMN protein responsible for degeneration of brain stem and spinal motor neurons. The paralogous SMN2 gene partially compensates full-length SMN protein production, mitigating the phenotype. Antisense oligonucleotide nusinersen (Spinraza^®^) enhances SMN2 gene expression. SMN is involved in RNA metabolism and biogenesis of microRNA (miRNA), key gene expression modulators, whose dysregulation contributes to neuromuscular diseases. They are stable in body fluids and may reflect distinct pathophysiological states, thus acting as promising biomarkers. Muscle-specific miRNAs (myomiRs) as biomarkers for clinical use in SMA have not been investigated yet. Here, we analyzed the expression of miR-133a, -133b, -206 and -1, in serum of 21 infantile SMA patients at baseline and after 6 months of nusinersen treatment, and correlated molecular data with response to therapy evaluated by the Hammersmith Functional Motor Scale Expanded (HFMSE). Our results demonstrate that myomiR serological levels decrease over disease course upon nusinersen treatment. Notably, miR-133a reduction predicted patients’ response to therapy. Our findings identify myomiRs as potential biomarkers to monitor disease progression and therapeutic response in SMA patients.

## 1. Introduction

Spinal muscular atrophy (SMA) is an autosomal recessive neuromuscular disorder characterized by selective loss of brainstem and spinal motor neurons (MNs), leading to progressive amyotrophic paralysis, respiratory deficiency, and, in more severe cases, death [1]. The clinical spectrum of SMA is heterogeneous and it is divided into five subtypes according to age of onset (from in utero to adult onset) and achieved motor milestones (from reduced or absent movements to very mild adult-onset phenotypes) [2,3]. The incidence of all types of SMA is approximately 1/10,000 live births, with a prevalence of 1-2/100,000 persons [4].

In about 96% of cases, SMA is caused by mutations or homozygous deletions involving the survival MN1 (SMN1) gene, located on chromosome 5q13.2. SMN1 gene is responsible for transcribing the majority of functional full-length (FL) SMN protein. There is a nearly identical SMN2 gene peculiar to humans, present in the same region of chromosome 5. SMN2 only differs from the SMN1 gene for a C- > T substitution that alters RNA splicing of exon 7. Consequently, the majority of SMN2 transcripts lack exon 7, resulting in a truncated nonfunctional protein. Only about 10–15% of the transcribed SMN2 protein is alternatively spliced to include exon 7, hence, encoding an amount of FL SMN protein insufficient to prevent disease [5,6]. The SMN2 copy number is variable across the SMA population, ranging from 0 to ≥ 4 copies; it represents a disease modifier where a higher SMN2 copy number is usually associated with a milder phenotype. However, there is partial overlap in the number of SMN2 copy numbers distributed among the different types of SMA patients [7,8,9,10,11].

Antisense oligonucleotide (ASO) and small molecules have been developed to alter the splicing pattern of SMN2 to increase the levels of transcription of FL SMN mRNA. The ASO nusinersen (Spinraza^®^) is the first approved treatment for SMA both in the USA [11] and Europe [12] and relies on this mechanism. It is designed to bind to a specific sequence in the pre-mRNA intron of exon 7, in the region occupied by the heterogeneous nuclear ribonucleoprotein (hRNP A1/2 proteins) that masks the intron splicing silencer N1 (ISS-N1) site. By displacing hRNP A1/2 from the ISS-N1 site, the ASO promotes the inclusion of exon 7, resulting in higher levels of functional SMN protein. ASOs do not cross the blood-brain barrier and must be administered intrathecally. Nusinersen has been demonstrated to improve motor function across SMA type I, II, and III (excluding the in utero- and adult-onset forms) [1].

Nowadays, clinical trials about small molecules SMN2 splicing modifiers (Risdiplam, Branaplam) are progressing, and some of these disease-modifying drugs are expected to soon be available for patient treatment. Finally, the first scAAV9 gene replacement therapy (Zolgensma^®^) has been recently approved by the FDA [13].

With the development of novel therapies, a current important medical need is the identification of non-invasive and non-clinical biomarkers to monitor disease progression and, particularly, therapeutic response. After nusinersen approval, such biomarkers have not been thoroughly investigated yet. Only plasma phosphorylated neurofilament heavy chain (pNF-H) has been identified as promising marker of disease activity/treatment response in children with SMA [14]. However, despite NF efficiency in monitoring neurodegeneration, they are not indicative of muscle health, which could also play a role in therapeutic response in SMA patients.

Growing evidence demonstrates the relevant role of microRNAs (miRNAs) as diagnostic and prognostic non-invasive biomarkers for motor neuron diseases (MNDs), such as amyotrophic lateral sclerosis (ALS) [15,16,17,18]. In SMA, miRNAs are among the most promising molecular biomarkers, due to the important role of SMN protein in RNA metabolism and miRNA biogenesis [19,20,21,22,23,24,25]. MiRNAs are small (~ 22 nucleotides) non-coding RNAs that regulate gene expression at post-transcriptional level [26]. They take part in different biological functions implicated in neuromuscular diseases, including SMA [27]. miRNAs are easy to detect, stable in body fluids and their expression levels reflect a distinct cell physiology state or damage to a specific tissue [21].

Catapano et al observed abnormal expression levels of miR-9, a neuron-related miRNA, and miR-206, a muscle-specific miRNA, in spinal cord, skeletal muscle and serum from transgenic mice, and in serum from SMA patients, suggesting that miRNAs could potentially serve as informative biomarkers to monitor disease progression, and response to ASO therapy in the SMA animal model [28].

Skeletal muscle-specific miRNAs, as miR-133a, -133b, -206 and -1, also known as myomiRs, are critical modulators of the myogenic program, by targeting a wide range of muscle genes [29]. Specifically, miR-133a and -133b promote myoblast proliferation [30], whereas miR-206 and -1 are involved in myoblast differentiation [31] cooperating in a regulatory circuit that mediates skeletal muscle regeneration. Recent studies demonstrated that myomiR expression is altered in skeletal muscle tissue of MND animals [32] and patients [33], suggesting a relevant role of these miRNAs in muscle degeneration and regeneration processes [17]. Previous studies investigated the differential expression levels of miR-206 and -133 in ALS serum compared to healthy control, suggesting a potential crucial role of these molecules in the early diagnosis and prognosis of the disease [15,34].

Currently, with the availability of nusinersen for SMA care, the possibility to collect biological samples from SMA patients treated with a disease-modifying drug represents a unique opportunity in the field of motor neuron diseases to identify reliable molecular factors, such as myomiRs, to monitor disease progression and therapeutic response.

In the present study, we investigated the expression profile of myomiRs in serum of pediatric SMA patients before and after 6 months of nusinersen treatment. Molecular data were correlated with clinical response to therapy, evaluated by the Hammersmith Functional Motor Scale Expanded (HFMSE). Our results revealed that expression levels of myomiRs decreased in SMA patients’ serum under nusinersen treatment, and that the reduction of miR-133a levels was associated with improvement in HFMSE values. We thus propose circulating myomiRs, particularly miR-133a, as possible non-invasive biomarkers of therapeutic effect of nusinersen in SMA patients.

## 2. Materials and Methods

### 2.1. Patients and Biological Samples

A cohort of consecutive 21 clinically defined SMA type II and III patients followed-up at the Developmental Neurology Unit, and genetically assessed at Unit of Medical Genetics and Neurogenetics, Fondazione IRCCS Istituto Neurologico Carlo Besta (Milan, Italy) was included in the study. Clinical assessment included the Hammersmith Functional Motor Scale Expanded (HFMSE). An improvement at the HFMSE assessment ≥ 3 points was considered clinically meaningful, as previously reported [35]. The study was performed in accordance with the ethical standards of the Declaration of Helsinki. The investigation and use of patients’ data for research purposes were approved by the Fondazione IRCCS Istituto Neurologico Carlo Besta research ethical committee in accordance with the Declaration of the World Medical Association (Project identification code 92/2019, 16 January 2019). Serum samples were obtained from peripheral blood after parental written consent, right before first nusinersen infusion (T0, baseline) and after 6 months of treatment (T6). Biological samples were stored at −80 °C in the Biobank of Fondazione IRCCS Istituto Neurologico Carlo Besta until use.

### 2.2. Quantitative Real-Time PCR to Determine miRNAs in Serum Samples

Total RNA was extracted with miRNeasy serum/plasma kit (Qiagen, Venlo, Netherlands) from 250 µL of serum. The RNA was retrotranscribed to cDNA using TaqMan MicroRNA Reverse Transcription Kits with primers specific for miR-133a, -133b, -206, -1 and -16; the latter used as endogenous control [36] was stably expressed in serum from patients at different conditions of treatments (as shown by standard deviation of Ct values < 0.5). cDNA aliquots corresponding to 15 ng total RNA were amplified by quantitative real time PCR in duplicate, with Universal PCR master mix and specific pre-designed TaqMan MicroRNA assays. miRNA levels were normalized to miR-16 and expressed as fold changes using the formula 2^−ΔCt^.

### 2.3. Statistical Analysis

Wilcoxon signed-ranked test was applied to analyze changes in miRNA expression levels before and after nusinersen treatment. Spearman’s correlation analysis was applied to verify whether serum levels of myomiRs, mir-133a, -133b, -206 and -1 correlated each other at baseline and 6 months after treatment. Logistic regression measured the relationship between changes in miRNA expression levels and clinical improvement. GraphPad Prism 8 (GraphPad Inc., La Jolla CA, USA) and R statistical environment (www.r-project.org) were used for statistical analyses. A *p* < 0.05 was considered statistically significant.

## 3. Results

### 3.1. Patient Cohort and Response to Therapy

A total of 21 consecutive SMA patients receiving nusinersen therapy at the Developmental Neurology Unit, Fondazione IRCCS Istituto Neurologico Carlo Besta (Milan, Italy) were recruited in the study. Patients’ clinical features at baseline are reported in Table 1. Out of 21 (eight males and 13 females), 16 patients were SMA II type, whereas five were SMA III type, with a mean age of 13.9 *±* 9.63 months at disease onset. The SMN2 gene copy number was determined for all the subjects and reported, together with the clinical features and motor function evaluations, in Table 2. The mean age at first infusion of nusinersen (baseline) was 5.18 *±* 3.42 years, with a HFMSE ranging from 1 (corresponding to very severe disease) to 62 (corresponding to mild disease) on a total score of 66 points (mean average 21.05 *±* 17.58). After 6 months of nusinersen therapy, 11 patients showed a clinically meaningful improvement at the HFMSE score (*≥* 3 points), seven presented an increase at the HFMSE score ranging from 1 to 2 points, 1 did not change, and 2 decreased their HFMSE scores by 1 and 2 points, respectively.

### 3.2. MyomiR Expression Reduction in Serum of SMA Patients during Nusinersen Treatment

**Figure 1:**
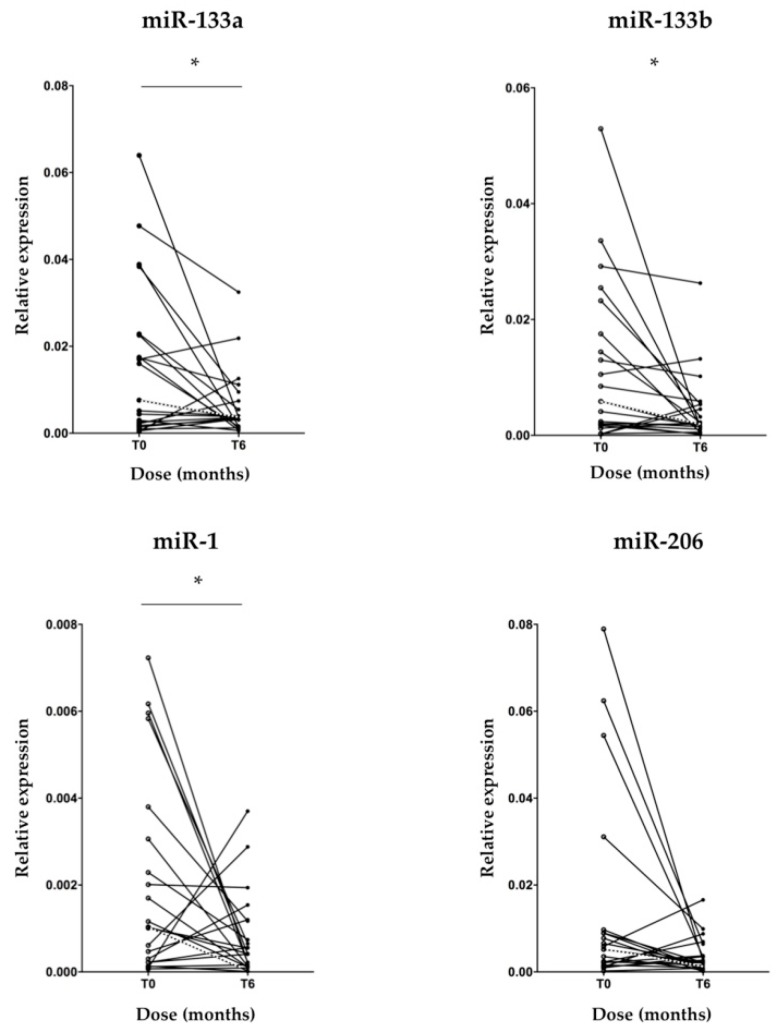
MyomiR expression reduction in serum of SMA patients after 6 months of nusinersen treatment. RT-PCR analysis of muscle-specific miRNAs in total RNA extracted from serum of 21 SMA patients demonstrated a significant reduction of miR-133a, -133b and -1 transcriptional levels during nusinersen treatment. A trend in reduction for miR-206 is shown. Each point represents miRNA expression level in serum of a patient at that time-point; trend lines show miRNA changes over time (solid lines). The medians of the myomiR expression levels at baseline and after 6 months of treatment are plotted accordingly for each miRNA (dotted lines). miRNA levels were normalized to miR-16 and expressed as fold changes using the 2^−ΔCt^ formula. Wilcoxon signed-ranked test, * *p* < 0.05.

**Figure 2:**
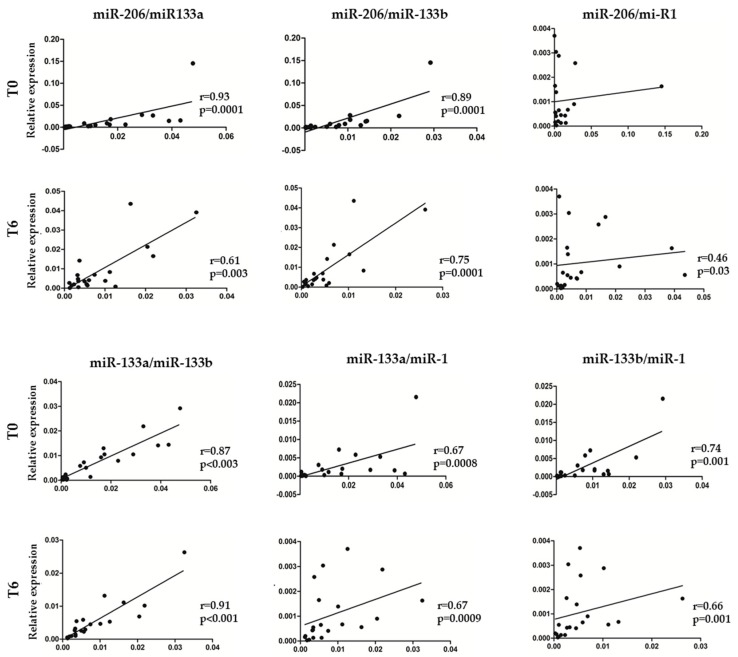
Spearman’s correlations between each myomiR expression in SMA patients during nusinersen treatment. Spearman’s correlation coefficients (r) were used to corroborate relationships between myomiR expression levels in serum of SMA patients at baseline (T0) and 6 months after treatment (T6). Coefficients major than 0.5 indicate a good positive correlation. *p* values (*p* < 0.05) and r (r ≥ 0.046) coefficients were assessed by Spearman test.

To identify miRNAs specifically expressed in skeletal muscle tissue as non-invasive biomarkers to monitor disease progression, we assessed the expression levels of miR-133a, -133b, -206 and -1 in patients’ serum samples *at* baseline and after 6 months of nusinersen treatment by real-time PCR. A significant decrease of miR-133a, -133b and -1 transcriptional levels after 6 months of therapy, compared to baseline values was found (Figure 1; *p* < 0.05). As regard to miR-206, a trend in reduction of the expression levels was observed, in line with the other tested myomiRs, but no significant difference compared to pre-treatment condition was found (Figure 1; *p* = 0.065). To verify whether the expression levels of mir-133a, -133b, -206 and -1, all belonging to the same miRNA cluster [30] correlated with each other, we performed a Spearman correlation analysis. Of interest, we found a significant positive correlation between miR-133a and -133b; miR-133a and -206; miR-133a and -1; miR-133b and -206; miR-133b and -1 serum levels, before and after 6 months of treatment (Figure 2; *p* < 0.05). The correlation between miR-206 and -1 was not significant at baseline (Figure 2).

### 3.3. Association between myomiR Expression Levels in Serum and Clinical Improvement of SMA Patients during Nusinersen Treatment

**Figure 3:**
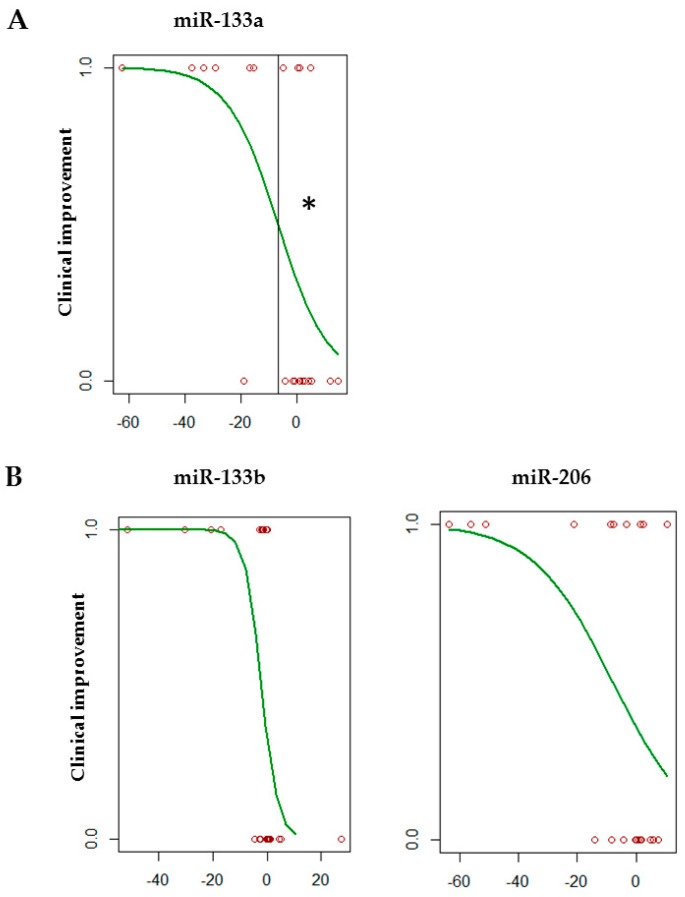
miR-133a reduction predicts clinical improvement of SMA patients during nusinersen treatment. Logistic regression analysis of miRNA differential expression and clinical response to therapy after 6 months of nusinersen treatment. Each circle represents miRNA expression level in serum of a patient after 6months of treatment. An improvement at the HFMSE score ≥ 3 was considered clinically meaningful (1.0 on y axis). A significant relationship for miR-133a reduction by 6.6 C_t_ values (inflection point) (**A**), and a positive trend for miR-133b and -206 are reported (**B**). * *p* < 0.05.

To determine whether myomiR differential expression in serum was able to predict response to therapy measured by HFMSE, logistic regression analysis was performed in SMA patients after 6 months of nusinersen treatment. A decrease of at least 6.6 cycle threshold (C_t)_ values (inflection point in the regression curve) in miR-133a expression levels was significantly associated with response to therapy, considered as an improvement at the HFMSE score ≥ 3 points (Figure 3; *p* < 0.05). Although there was a decrease of the expression levels of miR-133b and -206 in SMA patients with improved motor function after nusinersen treatment, there was no significant association between miRNA changes and clinical outcome (Figure 3; *p* = 0.184 and 0.119, respectively). The expression levels of miR-1 were not associated with HFMSE improvement (data not shown).

## 4. Discussion

In the recent years, the implementation of treatments able to modify disease course had revolutionized the approach to SMA, showing motor function improvements and prolonged survival [37]. Nusinersen is the first ever approved disease-modifying drug for 5q SMA type I, II and III; it acts by increasing levels of FL SMN protein. Non-clinical biomarkers of disease progression and response to treatment in SMA patients are lacking, albeit their identification would be largely supportive in clinical practice and in pharmacological clinical trials, including patients’ stratification. The discovery that myomiRs originating from peripheral tissues, such as damaged muscles, are detectable in human serum, corroborated their potential as easily accessible biomarkers for physiological and pathological muscle processes [17,18].

Growing evidence has shown a critical role of myomiRs in the development and maintenance of skeletal muscle [30,38]. Specifically, miR-133a, -133b, miR-206 and miR-1 are expressed under the control of myogenic transcription factors [39] and regulate skeletal myogenesis processes [40]. Their expression is enhanced during myoblast differentiation [39], and during muscle regeneration after injury in animal models [41].

Notably, serum myomiRs have been investigated in patients affected by neuromuscular diseases characterized by muscle degeneration and regeneration, such as Duchenne muscular dystrophy (DMD), where elevated levels of miR-133, -206 and -1, have been detected [42]. Similarly, miR-133a, -133b, miR-206 and -1 were up-regulated in plasma and serum samples from ALS patients [15,43], reflecting increased active myogenic processes in skeletal muscle biopsies [44], suggesting that changes in expression of myomiRs in serum samples of ALS patients might serve as a possible prognostic marker for the disease [18].

Here, we assessed for the first time the potential of selected myomiRs as non-invasive biomarkers to monitor disease progression and therapeutic response to nusinersen in pediatric SMA type II and type III patients. In the present study, we observed a significant decrease of miR-133a, -133b and -1 transcriptional levels after 6 months of nusinersen therapy, and a trend in reduction for miR-206, compared to pre-treatment condition. Notably, these miRNAs showed significant positive correlation among them indicating their synergistic expression in response to treatment. Interestingly, as above described, myomiRs have been reported to be up-regulated in serum from patients affected by DMD and ALS, which are diseases characterized by a significant muscle wasting, thus, further supporting a relationship between circulating myomiRs and pathophysiological changes occurring in muscle tissue, regardless the pathogenetic mechanism [17]. Based on these findings, our data on a marked myomiR reduction in SMA patients treated with nusinersen suggest an effect of the drug on muscle homeostasis that might be monitored by myomiR assessed in the serum. Since nusinersen is directly distributed within the central nervous system by intrathecal injection, and achieves therapeutic levels mostly in the target spinal cord tissue, we may hypothesize that its impact on skeletal muscles might be indirectly mediated by fostering SMN crucial function in motor neuron and neuromuscular junction maintenance [22,23,24], leading to reduced muscle denervation. A direct effect of nusinersen on skeletal muscle, where SMN depletion plays a crucial role in SMA muscle pathology, seems less likely, as shown by lack of significant increase in SMN2 full length mRNA in skeletal muscles of treated SMA compared to untreated [45].

The reduction of myomiR expression levels after treatments was also observed in previous studies using exon-skipping and morpholino oligomer-mediated dystrophin restoration therapies in DMD mice [42,46]. Similarly, Catapano and colleagues showed a decrease till normalization of miR-206 levels in serum of SMA I mice after oligonucleotide-based treatment, administered systemically, to increase FL SMN. However, in the same study, they did not find any significant difference in miR-206 serum level from 10 SMA patients compared to healthy controls [28]. Likewise, in our study, miR-206 did not show any significant change in expression compared to pre-treatment condition, even though it presented a trend toward reduction close to statistical significance.

Muscular dystrophies and motor neuron diseases rely on different pathogenic mechanisms but share a common feature which is muscular damage up to atrophy. Increased expression of myomiRs in serum samples of neuromuscular patients might reflect an unspecific compensatory response to cope with the muscular atrophy [42]. In this view, a decreased expression of myomiRs after treatment might be the result of an improvement of muscle conditions and performance. Pegoraro et al recently showed a significant decrease in levels of miR-133a, -133b, -206 and -1, after physical training in ALS patients; these findings were attributed to stabilization of skeletal muscle and neuromuscular junction (NMJ) of ALS patients [47].

In our cohort, 11 patients presented a clinical significant improvement of the motor function after 6 months of nusinersen treatment. We measured whether changes in myomiR expression levels were associated with clinical response to therapy and, notably, we identified that miR-133a reduction in serum of SMA patients after 6 months of nusinersen treatment was able to predict response to treatment measured by HFMSE. Despite serum miR-133b and -206 expression levels decreased in SMA patients who showed an improvement in motor function, there was no significant association between those miRNA changes and clinical outcome. This might be due to the relatively low number of patients enrolled in the analyses. Larger cohorts of patients, including also SMA type I and adults, are needed to confirm and further extend our results. Thus far, only neurofilament proteins (pNF-H) have been identified as promising marker of disease activity/treatment response in children with SMA type 1 [14,48]. However, *p*-NF levels in CSF have not proven to be a useful biomarker in adolescent and adult SMA type 2 and 3 patients [49,50,51]. NFs are exclusively expressed in neurons and released into extracellular fluids upon axon degeneration. As also reported by Kessler and colleagues, these findings might rely on the slower disease progression in SMA patients other than type 1, which might interfere with the detection of changes in CSF composition in a short evaluation period [52]. pNF-H role as biomarkers in SMA might be complementary to myomiRs, the former reflecting motor neuron damage and the latter informing about muscle tissue health during disease course. Subsequent evaluation of the detected myomiRs in SMA type 1 patients and correlation of their expression levels to pNF-H ones could lead to a better understanding of treatment response in the disease course.

Another limitation of this study is the lack of pediatric healthy controls, due to difficulty in collecting biological samples for the biobank in healthy children. This restricts the information we can gather about myomiR baseline expression levels; however, the performed paired statistical analysis in samples from treated patients compared to their own baseline (pre-treatment) allowed us to disclose significant variation in the expression of key myomiRs, obtaining novel informative data in SMA patients.

In summary, the current study demonstrates for the first time that expression levels of circulating myomiRs miR-133a, -133b, miR-206 and -1 decrease in SMA type II and III patients from baseline under nusinersen treatment, and that miR-133a level changes predicted motor function response to treatment.

Outcomes of this investigation support the potential role of serum myomiRs as non-invasive biomarkers to monitor disease progression and therapeutic response in SMA, laying the groundwork to individualize patient management in the clinical practice.

## 5. Conclusions

We observed for the first time a reduction in muscle-specific miRNAs under nusinersen treatment in pediatric SMA type II and III patients. A larger collection of data from SMA patients will be required to precisely establish a correlation between myomiR levels and clinical outcomes. The overall findings of this investigation highlight the relevance of miR-133a, -133b, -206 and -1 in the pathogenic processes underlying neuromuscular disorders, and support their potential as non-invasive biomarkers to monitor disease progression and measure the effectiveness of therapeutic interventions in SMA.

## Figures and Tables

**Table 1:** Clinical characteristics of the SMA patients at baseline.

SMA Features	Mean ± SD
Age (years)	5.18 ±3.42
Sex (M/F)	8/13
SMA type (II/III)	16/5
Disease duration (years)	4.04 ± 2.96
HFMSE	21.05 ± 17.58

**Table 2:** Clinical features and motor function evaluations of SMA patients before (T0) and after (T6) nusinersen treatment.

Patient	Gender	SMA Type	SMN2 Copy Number	AGE at ONSET (Months)	AGE at 1st INFUSION (Years/Months)	Baseline Motor Milestone	HFMSE at Baseline	HFMSE at 6th Month of Treatment	ΔHFMSE
Pt 01	M	II	3	10	3y8m	sitter	20	20	0
Pt 02	M	II	3	10	8y1m	sitter	13	11	−2
Pt 03	M	II	3	13	7y	sitter	14	15	+1
Pt 04	F	II	2,3	9	6y6m	sitter	12	14	+2
Pt 05	F	II	3	8	6y9m	sitter	8	9	+1
Pt 06	F	II	3	6	4y1m	sitter	22	32	+10
Pt 07	M	III	3	36	9y5m	sitter	57	61	+4
Pt 08	M	II	3	12	4y2m	walker	37	43	+6
Pt 09	F	III	3	36	13y10m	sitter	27	28	+1
Pt 10	F	II	3	8	1y11m	sitter	4	7	+3
Pt 11	M	II	3	12	2y	sitter	9	13	+4
Pt 12	F	II	3	10	4y5m	sitter	6	7	+1
Pt 13	M	III	2,3	18	3y5m	walker	43	47	+4
Pt 14	F	II	3	9	8y	sitter	19	21	+2
Pt 15	F	II	3	14	2y	sitter	30	33	+3
Pt 16	F	II	1,2	6	6y6m	sitter	3	2	−1
Pt 17	F	III	3	12	2y3m	walker	37	46	+9
Pt 18	M	II	3	8	8m	sitter	1	7	+6
Pt 19	F	III	3,4	36	9y10m	walker	62	63	+1
Pt 20	F	II	3	10	1y2m	sitter	14	17	+3
Pt 21	F	II	3	9	2y2m	sitter	4	8	+4
